# Training intensity influences left ventricular dimensions in young competitive athletes

**DOI:** 10.3389/fcvm.2022.961979

**Published:** 2022-10-06

**Authors:** Heidi Weberruß, Lisa Baumgartner, Frauke Mühlbauer, Nerejda Shehu, Renate Oberhoffer-Fritz

**Affiliations:** ^1^Department of Preventive Pediatrics, TUM Department of Sport and Health Sciences, Technical University of Munich, Munich, Germany; ^2^Department of Pediatric Cardiology and Congenital Heart Disease, German Heart Center Munich, Technical University of Munich, Munich, Germany

**Keywords:** cardiac remodeling, pre-participation screening, athlete’s heart, children, exercise

## Abstract

**Background:**

In young athletes, exercise causes changes in the heart that include growth in wall thickness and mass of the left ventricle and expansion of the heart’s chambers. The heart’s function is either preserved or enhanced, but this may change to the opposite over time.

**Objective:**

This study aimed to assess structural and functional cardiac adaptations in relation to exercise training time, intensity, and performance in young competitive athletes.

**Methods:**

A total of 404 children and adolescents (14.23 ± 2.0 years, 97 females) were enrolled in the Munich Cardiovascular Adaptations in Young Athletes Study (MuCAYA-Study). Eighty-five participants were examined two times a year. Two-dimensional echocardiography was performed to assess left ventricular structure and function. Training time and intensity was measured with the MoMo physical activity questionnaire, maximum aerobic capacity by cardiopulmonary exercise testing, and strength with the handgrip strength test.

**Results:**

Maximum aerobic capacity significantly influenced interventricular septal thickness in diastole. Training intensity significantly influenced left ventricular internal diameter in diastole and systole, and left ventricular mass indexed to body surface area. Within one year, interventricular wall thickness, relative wall thickness and left ventricular mass, indexed to body surface area and height, increased significantly. Training intensity and aerobic capacity contributed to cardiac adaptations in young competitive athletes, as represented by altered structural parameters but preserved cardiac function. Within a year, however, structural changes and a decline in diastolic performance were observed within the longitudinal sub-sample.

**Conclusion:**

Our results confirm the hypothesis that cardiac adaptations to exercise occur at a young age. Cardiac adaptation in our cohort was influenced by exercise intensity and maximum aerobic capacity.

## Introduction

The heart adapts to exercise beginning at a young age. In young competitive athletes, left ventricular (LV) diameter (1–6), volume (7–10), wall thickness, and mass are increased (1, 3, 4, 6, 10–12). Binnetoglu et al. (13) reported concentric remodeling in 15.7% and concentric hypertrophy in 14.3% of young athletes at a mean age of 12.2 ± 0.8 years. Other authors observed eccentric LV remodeling in endurance athletes and concentric LV remodeling in power athletes (5, 14). The right ventricle (RV) and both atria are also affected by physical training (1, 3, 8, 15, 16). Cardiac function, however, is not affected at a young age (2, 7, 8, 15). In adult athletes, on the contrary, exercise-induced ventricular arrhythmia, atrial dilatation, and tricuspid regurgitation can be observed as a negative consequence of intense exercise (17–21).

As training loads and levels of competitiveness increase over an athlete’s career (3, 15), it is essential to follow young athletes regularly to monitor their cardiac adaptations and not to miss the point when positive adaptations may develop in an adverse direction (22, 23).

Therefore, this study investigated the cardiac structure and function by two-dimensional echocardiography in young competitive athletes in relation to training time per week, training intensity, and maximal exercise performance. We hypothesized that young competitive athletes show an altered cardiac structure and function compared to reference values relative to their training volumes and intensity.

## Materials and methods

This work was part of the Munich Cardiovascular Adaptations in Young Athletes Study (MuCAYA-Study) (24), which was conducted from September 2018 to September 2020 at the Chair of Preventive Pediatrics, TUM Department of Sport and Health Sciences, Technical University of Munich (TUM). This study was approved by the local ethics committee (301/18S) and is in line with the Declaration of Helsinki (2013). Informed consent was obtained from all participants and their legal guardians.

### Subjects

465 young competitive athletes (7–18 years) visited our department for a pre-participation screening. Only participants who regularly participated in competitions and trained ≥3 h/week were included in further analyses, as this amount of training time has been shown to elicit cardiac adaptations (25, 26). Furthermore, the following inclusion criteria were applied: age 7–18 years, informed consent by children and/or legal guardians, no acute infection, no acute orthopedic injury, medical clearance for cardiopulmonary exercise testing. Participants who did not participate in competitions, did not exercise regularly for ≥3 h/week, or did not meet all inclusion criteria were excluded from participating in the study.

According to the main type of sports, participants were classified into four categories, as defined by Pelliccia et al. (26)—namely mixed category (isotonic and isometric components, moderate cardiac remodeling, e.g., soccer or basketball), endurance (isotonic > isometric, pronounced cardiac remodeling, e.g., cycling or rowing), power (isometric > isotonic, less cardiac remodeling, e.g., weight lifting or boxing) and skill (low isotonic and isometric components, minor cardiac remodeling, e.g., golf or sailing). Participants with one visit were included in the cross-sectional analysis (V1). A longitudinal sub-sample analysis included *n* = 85 participants (eight girls) with two visits within one year (V1 vs. V2). According to McClean et al. (3), who found significantly increased echocardiographic parameters in subjects ≥14 years compared to subjects <14 years, the sample was sub-divided into these two age groups. The rationale lies in significant pubertal landmarks at this age (27–30). Tanner stages were not assessed due to ethical considerations and child protection.

### Anthropometry

Body height and mass were measured without shoes and standing upright. Body height was registered to the next 0.1 cm and body weight to the next 0.1 kg (seca 799, seca GmbH&Co.KG, Hamburg, Germany). Additionally, body mass index (BMI, in kg/m^2^), waist-to-hip ratio (WHR), and waist-to-height ratio (WHtR) were calculated. Standardized z-scores for body height, BMI, WHR, and WHtR were compared to German reference values (31). Body surface area (BSA, in m^2^) was calculated according to Dubois and Dubois (32).

### Heart rate, blood pressure, and pulse wave analysis

Resting heart rate (HR) and peripheral systolic and diastolic blood pressure (SBP/DBP) were measured oscillometrically in a supine position after 10 min of rest (Mobil-O-Graph^®^, I.E.M., Stolberg, Germany). An appropriate cuff was placed on the participants’ left arm. Central systolic blood pressure (cSBP) and pulse wave velocity (PWV) were determined with the ARCSolver pulse wave analysis algorithm (AIT, Austrian Institute of Technology GmbH, Vienna, Austria) (33). The method is validated against invasive catheter measurements of cSBP [*r*^2^ = 0.899, *p* < 0.0001, (34), *r*^2^ = 0.97, *p* < 0.001, (35)] and PWV [*r*^2^ = 0.81, *p* < 0.001, (36)], as well as against non-invasive measurements with the SphygmoCor [*r*^2^ = 0.532, *p* < 0.05 (37)]. Standardized z-scores were compared to German reference values (38, 39).

### Cardiopulmonary exercise testing

After medical clearance, participants performed a cardiopulmonary exercise test (CPET) on an ergobike (Lode Corival, Lode B.V., Groningen, Netherlands) with spirometric measurement (Ergostik, Geratherm Respiratory GmbH, Bad Bissingen, Germany) of maximum aerobic capacity (VO_2peak_), and a 12-lead ECG (CARDIOVIT CS-200 Office, SCHILLER AG, Baar, CH). A modified Godfrey protocol was followed (40). After 2 min of rest, participants started cycling with an initial load of 50% of their body weight. The incline was chosen to reach 4–5 Watt/kg body mass within 6–12 min at a cadence of 60–80 rpm (41, 42). Maximum heart rate (1/min), maximum workload (Watt), relative maximum workload (Watt/kg), and relative VO_2peak_ (ml/min/kg) were assessed.

### Handgrip strength

Participants’ handgrip strength (HGS) was assessed in a seated position with the upper body upright, shoulders abducted at 10°, both elbows flexed at 90°, and the forearm in a neutral position, according to the standardized recommendations by the American Society of Hand Therapists (ASHT) (43). As HGS is closely correlated with overall muscular strength, it was applied as a surrogate parameter for muscular strength in this study (44). The handgrip dynamometer (SAEHAN Hydraulic Hand Dynamometer SH5001, SAEHAN Corporation, Masan, South Korea) was pushed at maximum strength, alternately three times with the right hand and three times with the left hand. For further calculations, the maximum attempt was applied in relation to the participants’ body mass (45). In a study on children, HGS showed a high correlation with the 1-repetition maximum bench press test (*r* = 0.79, *p* < 0.01; *R*^2^ = 0.621, *p* < 0.01) (46).

### Physical activity questionnaire

Participants’ training history over the years, exercise training time (h/week), and intensity in metabolic equivalents (METs) were assessed with the self-reported MoMo (Motorik Modul) physical activity questionnaire (47). Participants reported how much they exercised (min/week) and at which intensity rated on a three-item scale (low, moderate, intense). According to this, training time in h/week was calculated and adjusted for a factor depending on how many months a year the sport was performed. An intensity index was derived based on the calculation of METs. One MET refers to the body’s oxygen consumption of 3.5 ml O_2_/min/kg when sitting at rest. In comparison, the body consumes 8.8 METs while playing soccer at moderate intensity. For a soccer game of 90 min, this refers to an intensity of 792 MET-minutes or 13.2 MET-hours, respectively. MET-values for different types of sports were provided by Schmidt et al., Ridley et al., and Ainsworth et al. (47–49). The questionnaire’s Kappa coefficient is 0.66, and the intraclass correlation coefficient is 0.68 (50).

### Echocardiography

Transthoracic echocardiographic measurements were performed to assess LV dimensions, such as LV internal diameter in diastole (LVIDd) and systole (LVIDs), interventricular septal thickness in diastole (IVSd), and LV posterior wall thickness in diastole (LVPWd) in M-mode. Relative wall thickness (RWT) was calculated according to Lang et al. (51). Standardized z-scores for LVIDd, LVIDs, IVSd, and LVPWd were derived according to reference values by Pettersen et al. (52). LV mass (LVM) was calculated according to Devereux and Reicheck (53) and presented as indexed values relative to BSA (LVM/BSA) and body height (LVM/height). For the differentiation between LV eccentric hypertrophy (RWT ≤ 0.42 and LVM/m^2.7^ > P95) and concentric hypertrophy (RWT > 0.42 and LVM/m^2.7^ > P95), reference intervals for indexed LVM (LVM/m^2.7^) were calculated according to Khoury et al. (54). LV systolic function was assessed by ejection fraction (EF), measured in B-mode (biplane Simpson’s method), and fractional shortening (FS), measured in M-mode. LV diastolic function was indirectly assessed by the ratio of mitral E- and A-wave, measured via pulsed-wave Doppler at a standardized position with the sample volume at the tips of the open mitral valve leaflets. All measurements were performed with a GE VIVID 7 Dimension ultrasound system (GE Healthcare, Horten, Norway) and off-line analyses with dedicated software (ECHOPAD Software, GE Healthcare, Horten, Norway). Two experienced pediatric cardiologists performed all measurements and off-line analyses.

### Statistics

The statistical analysis was performed with SPSS statistical software, version 25 (IBM, Chicago, IL, USA). For the cross-sectional sample (V1), descriptive data were calculated for the entire study population, for boys and girls, separately, and for boys and girls within the two age groups (<14 years and ≥14 years). Sex differences within the overall sample and differences between the two age groups were tested by independent *t*-tests. Standardized z-scores were compared to reference values via a one-sample *t*-test.

The influence of training time per week, training intensity, VO_2peak_, and HGS on echocardiographic parameters was examined via linear multiple regression analysis. Quintiles (Q1–Q5) were calculated for boys and girls separately to compare different groups regarding training time, training intensity, VO_2peak_, and HGS. As the data did not meet the assumptions for a one-way non-parametric analysis of covariance (ANCOVA), Quade’s non-parametric ANCOVA with Tukey *post hoc* correction was applied (55), controlling for sex, age, BSA (except for the analysis of LVM/BSA), SBP, and training history in years. Differences between athletes performing endurance and power sports were analyzed by an independent *t*-test (parametric data) or Mann–Whitney U-Test (non-parametric data).

The longitudinal sub-sample (V1 vs. V2) was compared via the dependent *t*-test for parametric data and the Wilcoxon Matched-Pairs Test for non-parametric data, respectively. Significant results were reported at a *p*-value < 0.05.

## Results

**FIGURE 1:**
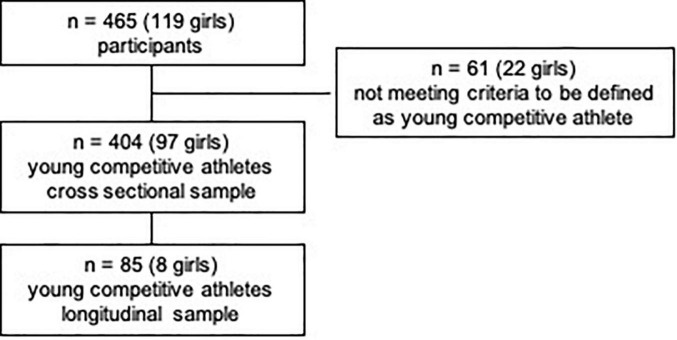
Study flow chart.

**FIGURE 2:**
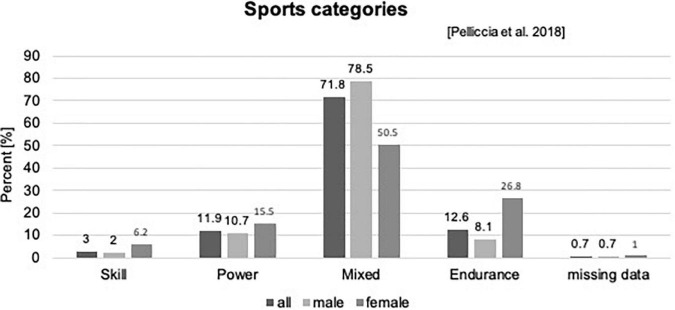
Sports categories according to Pelliccia et al. (26).

Out of the 465 (7–18 years) children and adolescents, who performed a pre-participation screening at our department, *n* = 404 (97 girls) matched the criteria to be defined as a young competitive athlete, regularly training ≥3 h/week (25, 56), Figure 1. Participants performed 32 different types of sports in an organized sports club setting with regular competitions. We categorized sports according to Pelliccia et al. (26): 71.8% of the types of sports performed could be assigned to the mixed category (isotonic and isometric components, along with moderate cardiac remodeling); 12.6% were predominantly endurance (isotonic > isometric, pronounced cardiac remodeling), 11.9% were predominantly power (isometric > isotonic, less cardiac remodeling), and 3% were skill types of sports (isotonic and isometric, little cardiac remodeling, Figure 2 and Supplementary Table 1). The average training history, e.g., the time since participants had started performing competitive sports, was 3.6 ± 2.5 years. The average training time was 8.40 ± 3.59 h/week at an intensity of 78.35 ± 33.70 MET-h/week (Table 1).

**TABLE 1:** Participants’ anthropometric characteristics, data on heart rate, blood pressure, and pulse wave analysis, as well as data on physical performance and training time.

Anthropometry		All		Males		Females	*P*-value
	n	mean ± SD	n	mean ± SD	n	mean ± SD	
Age [years]	404	14.23 ± 2.00	307	14.27 ± 1.99	97	14.11 ± 2.04	0.496
Body height [cm]	403	167.14 ± 14.41	306	168.64 ± 15.02	97	162.43 ± 11.12	**< 0.001**
Body height z-score	403	0.30 ± 1.15	306	0.29 ± 1.15	97	0.32 ± 1.15	0.836
Body mass [kg]	403	56.62 ± 14.78	306	57.65 ± 15.52	97	53.36 ± 11.62	**0.004**
BMI [kg/m^2^]	403	19.87 ± 2.75	306	19.84 ± 2.75	97	19.96 ± 2.73	0.716
BMI z-score	403	0.04 ± 0.80	306	0.04 ± 0.80	97	0.03 ± 0.80	0.938
WHR z-score	381	−0.40 ± 1.07	293	−0.47 ± 1.07	88	−0.17 ± 1.04	**0.018**
WHtR z-score	381	−0.39 ± 0.71	293	−0.40 ± 0.71	88	−0.38 ± 0.72	0.812
BSA [m^2^]	403	1.61 ± 0.28	306	1.64 ± 0.29	97	1.55 ± 0.22	**< 0.001**

**Heart rate and blood pressure**		**All**		**Males**		**Females**	***P*-value**
	**n**	**mean ± SD**	**n**	**mean ± SD**	**n**	**mean ± SD**	

HR [1/min]	403	65.15 ± 10.11	307	64.70 ± 9.51	96	66.59 ± 11.76	0.110
SBP [mmHg]	403	116.15 ± 8.96	307	117.23 ± 9.26	96	112.71 ± 6.88	**< 0.001**
SBP z-score	402	0.23 ± 0.89	306	0.25 ± 0.91	96	0.15 ± 0.83	0.325
DBP [mmHg]	403	63.49 ± 6.21	307	63.50 ± 6.29	96	63.46 ± 6.00	0.960
DBP z-score	402	−0.59 ± 0.92	306	−0.60 ± 0.92	96	−0.57 ± 0.93	0.807

**Pulse wave analysis**		**All**		**Males**		**Females**	***P*-value**
	**n**	**mean ± SD**	**n**	**mean ± SD**	**n**	**mean ± SD**	

PWV [m/s]	403	4.90 ± 0.44	307	4.94 ± 0.46	96	4.76 ± 0.34	**< 0.001**
PWV z-score	400	0.49 ± 1.35	305	0.51 ± 1.38	95	0.45 ± 1.28	0.720
cSBP [mmHg]	403	105.13 ± 10.31	307	105.85 ± 10.65	96	102.84 ± 8.81	**0.013**
cSBP z-score	400	0.39 ± 1.29	305	0.41 ± 1.30	95	0.35 ± 1.24	0.719

**Cardiopulmonary exercise test**		**All**		**Males**		**Females**	***P*-value**
	**n**	**mean ± SD**	**n**	**mean ± SD**	**n**	**mean ± SD**	

Maximum HR [1/min]	361	186.58 ± 11.05	282	186.42 ± 11.09	79	187.14 ± 10.99	0.609
Maximum power output [Watt]	363	254.07 ± 76.21	282	264.78 ± 79.43	81	216.80 ± 48.08	**< 0.001**
Relative power output [Watt/kg]	363	4.47 ± 0.63	282	4.60 ± 0.57	81	4.03 ± 0.64	**< 0.001**
Relative VO_2peak_ [ml/min/kg]	358	44.23 ± 7.49	278	46.08 ± 6.66	80	37.78 ± 6.61	**< 0.001**

**Handgrip strength**		**All**		**Males**		**Females**	***P*-value**
	**n**	**mean ± SD**	**n**	**mean ± SD**	**n**	**mean ± SD**	

Maximum HGS/body mass	366	0.53 ± 0.10	282	0.53 ± 0.10	84	0.50 ± 0.08	**< 0.001**

**Physical activity questionnaire**		**All**		**Males**		**Females**	***P*-value**
	**n**	**mean ± SD**	**n**	**mean ± SD**	**n**	**mean ± SD**	

Days of physical activity/week	404	5.19 ± 1.20	307	5.28 ± 1.17	97	4.92 ± 1.24	**0.011**
Main sport: training/week [h]	403	7.96 ± 3.59	306	7.94 ± 3.53	97	7.73 ± 4.14	0.464
Sports club activity: training/week [h]	404	8.40 ± 3.59	307	8.41 ± 3.49	97	8.36 ± 3.81	0.900
Main sport: MET-hours/week	400	72.23 ± 36.39	304	73.93 ± 33.35	96	66.83 ± 44.46	0.152
Sports club activity: MET-hours/week	402	78.35 ± 33.70	306	79.23 ± 30.98	96	75.53 ± 41.26	0.419

BMI, body mass index; WHR, waist-to-hip ratio; WHtR, waist-to-height ratio; BSA, body surface area; HR, heart rate; SBP, systolic blood pressure; DBP, diastolic blood pressure; PWV, pulse wave velocity; cSBP/cDBP, central SBP/DBP; HR, heart rate; VO_2peak_, maximum oxygen capacity; HGS, hand grip strength; MET, metabolic equivalent. The meaning is that these values are significant results as indicated by a p-value of < 0.05.

### Anthropometry

The participants’ mean age was 14.23 ± 2.00 years. Boys and girls differed significantly in body height and mass, WHR, and BSA. Anthropometric data are displayed in Table 1.

### Heart rate, blood pressure, and pulse wave analysis

There were no significant sex differences in participants’ heart rates (boys 64.7 ± 9.51/min vs. girls 66.59 ± 11.76/min, *p* = *0.110)*. Boys had a significantly higher SBP and PWV (*p* < *0.001*) and cSBP (*p* = *0.013*) than girls (Table 1). Compared to reference values, boys had significantly higher z-scores for SBP (0.25 ± 0.91), cSBP (0.41 ± 1.3), and PWV (0.51 ± 1.38, *p* < *0.001* for all) and a lower DBP (-0.60 ± 0.92, *p* < *0.001*). In girls, cSBP (0.35 ± 1.24, *p* = *0.007*) and PWV (0.45 ± 1.28, *p* = *0.001*) were significantly higher, and DBP was significantly lower (-0.57 ± 0.93, *p* < *0.001*) than observed in the reference population.

### Cardiopulmonary exercise testing, handgrip strength, and physical activity

Boys performed significantly better in CPET testing (+14.1% W/kg and +22% VO_2peak_) and HGS (+6%, for all *p* < *0.001*) than girls. Regarding training time and training intensity, no significant sex differences were observed. Boys trained 8.41 ± 3.49 h/week at an intensity of 79.23 ± 30.98 MET-h/week and girls 8.36 ± 3.81 h/week at an intensity of 75.53 ± 41.26 (Table 1).

### Echocardiographic parameters

A total of 391 competitive athletes underwent 2D transthoracic echocardiography. None of the athletes presented with LV hypertrophy (LVPWd > 12 mm). Eccentric hypertrophy was found in 54.1% of males and 45.4% of females, and concentric hypertrophy in 5.5% of males and 8.2% of females. In 34.9% of males and 43.3% of females, normal LV geometry was observed.

**FIGURE 3:**
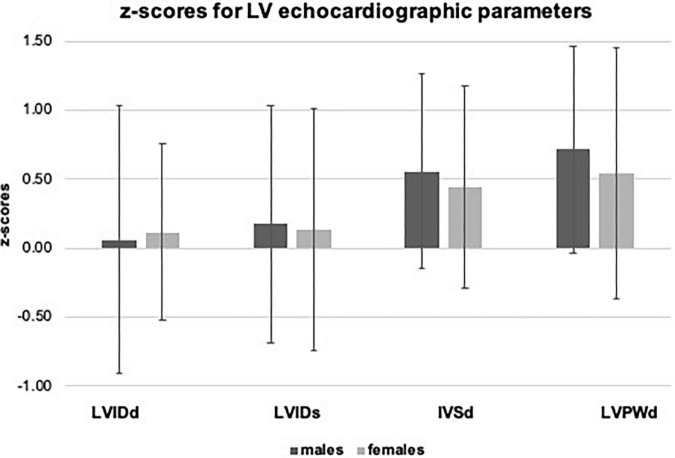
z-scores for echocardiographic left ventricular parameters.

In the overall sample, we observed significantly higher values for z-scores of LVIDs (0.16 ± 0.87), IVSd (0.53 ± 0.72), and LVPWd (0.67 ± 0.79, *p* < *0.001* for all). Significant sex differences with higher values in boys were observed for absolute values of LVIDd (+ 5.3%), LVIDs (+ 6.1%), IVSd (+ 7.9%), and LVPWd (+ 9.0%), but not for corresponding z-scores (*p* > *0.05*). LVM, indexed for BSA and body height, was significantly higher in males compared to females (LVM/BSA + 15.3%, LVM/body height + 17.6%, *p* < *0.001*). Results are displayed in Table 2, and age- and sex-specific z-scores for LVIDd, LVIDs, IVSd, and LVPWd are displayed in Figure 3.

**TABLE 2:** Results of echocardiographic data for all participants and separately for male and female athletes.

2D transthoracic echocardiography		All		Males		Females	*P*-value
	n	mean ± SD	n	mean ± SD	n	mean ± SD	
EF [%]	389	66.61 ± 5.77	294	66.41 ± 5.75	95	67.23 ± 5.83	0.231
FS [%]	388	37.10 ± 4.62	293	36.96 ± 4.52	95	37.54 ± 4.91	0.293
LVIDd [mm]	389	48.16 ± 4.89	294	48.76 ± 5.00	95	46.29 ± 4.04	**< 0.001**
LVIDd z-score	388	0.08 ± 0.90	293	0.06 ± 0.97	95	0.12 ± 0.64	0.546
LVIDs [mm]	388	30.28 ± 3.97	293	30.71 ± 4.02	95	28.95 ± 3.53	**< 0.001**
LVIDs z-score	387	0.16 ± 0.87	292	0.17 ± 0.86	95	0.13 ± 0.88	0.670
IVSd [mm]	391	8.56 ± 1.44	296	8.72 ± 1.46	95	8.08 ± 1.26	**< 0.001**
IVSd z-score	390	0.53 ± 0.72	295	0.56 ± 0.71	95	0.45 ± 0.73	0.179
LVPWd [mm]	389	8.07 ± 1.39	294	8.24 ± 1.34	95	7.56 ± 1.41	**< 0.001**
LVPWd z-score	388	0.67 ± 0.79	293	0.71 ± 0.75	95	0.54 ± 0.91	0.094
Relative wall thickness	389	0.35 ± 0.05	294	0.35 ± 0.05	95	0.34 ± 0.06	0.160
LVM/BSA [g/m^2^]	388	105.04 ± 20.35	293	108.56 ± 19.98	95	94.18 ± 17.53	**< 0.001**
LVM/body height [g/m]	388	101.30 ± 25.00	293	105.16 ± 25.41	95	89.39 ± 19.42	**< 0.001**
LVM/body height [g/m^2.7^]	388	42.12 ± 8.14	293	43.08 ± 8.10	95	39.16 ± 7.58	**< 0.001**
E/A	278	2.46 ± 2.16	205	2.40 ± 2.10	73	2.61 ± 2.32	0.474

EF, ejection fraction; FS, fractional shortening; LVIDd, left ventricular internal diameter in diastole; LVIDs, left ventricular internal diameter in systole; IVSd, interventricular septal thickness in diastole; LVPWd, left ventricular posterior wall thickness in diastole; RWT, relative wall thickness; LVM/BSA, left ventricular mass/body surface area; LVM/body height, left ventricular mass/body height; E/A, ratio of mitral E- and A-wave. The meaning is that these values are significant results as indicated by a p-value of < 0.05.

In linear multiregression analysis, controlled for sex, age, BSA, SBP, and training history in years, training intensity (MET-h/week) significantly influenced LVIDd (β = *0.07, p* = *0.04)*, LVIDs (β = *0.10, p* = *0.01)*, and LVM/BSA (β = *0.10, p* = *0.05)*. Sex, BSA, and training intensity predicted LVIDd *[R^2^* = *0.610, F* (6, 361) = *94.146, p* < *0.001]* and LVIDs *[R^2^* = *0.495, F* (6, 360) = *58.747, p* < *0.001]*. Sex, age, SBP, and training intensity explained 20% of the variance in LVM/BSA *[R^2^* = *0.200, F* (5, 362) = *16.991, p* < *0.001].*

Interventricular septal thickness in diastole (IVSd) and LVM/BSA were significantly associated with VO_2peak_ *(IVSd:* β = *0.11, p* = *0.05; LVM/BSA:* β = *0.12, p* = *0.04)*; sex, BSA, training history, and VO_2peak_ explained 26.9% of the variance in IVSd *[R^2^* = *0.269, F*(6, 329) = *20.141, p* < *0.001] and 21.9% in LVM/BSA [R^2^* = *0.219, F*(5, 328) = *18.366, p* < *0.001]*. Regarding HGS, no significant influence on echocardiographic parameters was observed. Significant results of the linear multi-regression analysis are displayed in Table 3.

**TABLE 3:** Results of linear multi-regression analysis controlled for sex, age, body surface area, systolic blood pressure, training history, and training intensity or maximum aerobic capacity, respectively.

Training intensity [MET-hours/week]	LVIDd *R*^2^ = 0.610 (*p* < 0.001)			
	
	β ± SE	β_standardized_	*P*	95% CI
Constant	25.07 ± 2.27		< 0.001	20.61–29.54
**Sex [boys vs. girls]**	−**1.10 ± 0.39**	−**0.10**	**0.01**	−**1.85 to 0.34**
Age [years]	−0.13 ± 0.14	−0.05	0.35	−0.39–0.14
**BSA [m^2^]**	**13.47 ± 0.96**	**0.77**	**< 0.001**	**11.59**–**15.35**
SBP [mmHg]	0.03 ± 0.02	0.05	0.22	−0.02–0.06
Training history [years]	−0.05 ± 0.07	−0.03	0.46	−0.18–0.08
**Training intensity [MET-hours/week]**	**0.01 ± 0.01**	**0.07**	**0.04**	**0.00**–**0.02**

**Training intensity [MET-hours/week]**	**LVIDs *R*^2^ = 0.495 (*p* < 0.001)**			
	
	**β ± SE**	**β_standardized_**	***P***	**95% CI**

Constant	14.68 ± 2.13		< 0.001	10.49–18.86
**Sex [boys vs. girls]**	−**0.92 ± 0.36**	−**0.10**	**0.01**	−**1.63 to** −**0.21**
Age [years]	−0.03 ± 0.13	−0.01	0.82	−0.28–0.22
**BSA [m^2^]**	**9.74 ± 0.90**	**0.67**	**< 0.001**	**7.97**–**11.50**
SBP [mmHg]	0.00 ± 0.02	−0.01	0.86	−0.04–0.03
Training history [years]	0.01 ± 0.06	0.01	0.88	−0.12–0.13
**Training intensity [MET-hours/week]**	**0.01 ± 0.01**	**0.10**	**0.01**	**0.00**–**0.02**

**Training intensity [MET-hours/week]**	**LVM/BSA *R*^2^ = 0.200 (*P* < 0.001)**			
	
	**β ±** **SE**	**β_standardized_**	***P***	**95% CI**

Constant	32.28 ± 13.36		0.016	6.01–58.56
**Sex [boys vs. girls]**	−**12.07 ± 2.28**	−**0.26**	**< 0.001**	−**16.56 to** −**7.58**
**Age [years]**	**1.90 ± 0.54**	**0.18**	**< 0.001**	**0.84**–**2.96**
**SBP [mmHg]**	**0.37 ± 0.12**	**0.16**	**0.002**	**0.14–0.59**
Training history [years]	0.50 ± 0.40	0.06	0.21	−0.28–1.28
**Training intensity [MET-hours/week]**	**0.06 ± 0.03**	**0.10**	**0.05**	**0.00**–**0.12**

**VO_2peak_ [ml/min/kg]**	**IVSd *R*^2^ = 0.269 (*p* < 0.001)**			
	
	**β ± SE**	**β_standardized_**	***P***	**95% CI**

Constant	3.54 ± 1.00		< 0.001	1.58–5.50
**Sex [boys vs. girls]**	−**0.37 ± 0.19**	−**0.11**	**0.05**	−**0.74 to** −**0.01**
Age [years]	−0.05 ± 0.06	−0.07	0.41	−0.17–0.07
**BSA [m^2^]**	**2.40 ± 0.42**	**0.46**	**< 0.001**	**1.58–3.21**
SBP [mmHg]	0.01 ± 0.01	0.04	0.47	−0.01–0.02
**Training history [years]**	**0.07 ± 0.03**	**0.12**	**0.01**	**0.01**–**0.13**
**VO_2peak_ [ml/min/kg]**	**0.02 ± 0.01**	**0.11**	**0.05**	**0.00**–**0.04**

**VO_2peak_ [ml/min/kg]**	**LVM/BSA *R*^2^ = 0.219 (*p* < 0.001)**			
	
	**β ± SE**	**β_standardized_**	***P***	**95% CI**

Constant	21.89 ± 14.30		< 0.001	1.58–5.50
**Sex [boys vs. girls]**	−**11.01 ± 2.64**	−**0.24**	**< 0.001**	−**16.25** to −**5.86**
**Age [years]**	**1.88 ± 0.55**	**0.18**	**0.001**	**0.80**–**2.97**
**SBP [mmHg]**	**0.38 ± 0.12**	**0.17**	**0.001**	**0.15**–**0.61**
Training history [years]	0.09 ± 0.42	0.11	0.832	−0.73–0.91
**VO_2peak_ [ml/min/kg]**	**0.31 ± 0.15**	**0.12**	**0.04**	**0.01**–**0.60**

BSA, body surface area; SBP, systolic blood pressure; MET, metabolic equivalent; VO_2peak_, maximum oxygen capacity; LVIDd, left ventricle internal diameter in diastole; LVIDs, left ventricle internal diameter in systole; LVM, left ventricular mass; IVSd, interventricular septal thickness in diastole. The meaning is that these values are significant results as indicated by a p-value of < 0.05.

**FIGURE 4:**
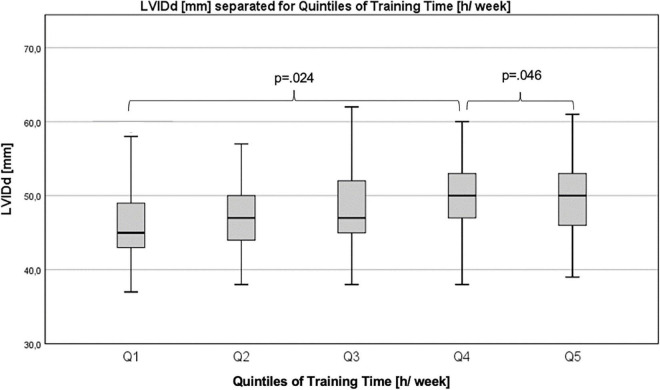
Differences in left ventricular internal diameter in diastole between quintiles of training time.

Differences in echocardiographic parameters between quintiles (Q1–Q5) for training time, training intensity, VO_2peak_, and HGS were examined using Quade’s non-parametric ANCOVA. Ranges for quintiles can be found as Supplementary Table 2. LVIDd differed significantly for quintiles of training time (h/week) between Q1 and Q4 (Q1: 46.26 ± 5.21 mm vs. Q4: 49.26 ± 4.91 mm, *p* = *0.024*) and between Q4 and Q5 (Q4: 49.26 ± 4.91 mm vs. Q5: 49.79 ± 4.87 mm, *p* = *0.046*; Figure 4). There were no significant differences between quintiles for any of the other parameters.

### Comparison of boys and girls <14 years and ≥14 years

Boys and girls < 14 years did not differ significantly in anthropometric parameters, heart rate, BP and PWV, training time, training intensity, and HGS. Z-scores for LVIDd and LVIDs were significantly higher in boys vs. girls < 14 years (LVIDd: 0.34 ± 0.56 vs. 0.07 ± 0.62, *p* = *0.016*; LVIDs: 0.28 ± 0.64 vs. -0.12 ± 0.96, *p* = *0.003*). The difference did not persist in the older age group. Absolute values for LVIDd, LVIDs, IVSd, and LVPWd were higher in boys vs. girls ≥14 years. LVM/BSA was higher in boys than in girls in both age groups (see Supplementary Table 3).

### Comparison of endurance athletes vs. power athletes

Endurance athletes had a significantly lower DBP than power athletes (DBP: 62.06 ± 5.81 vs. 66.08 ± 8.08 mmHg, *p* = *0.006*; DBP z-score: -0.81 ± 0.82 vs. -0.23 ± 1.20, *p* = *0.007*). Among echocardiographic parameters, E/A was higher in endurance athletes (2.87 ± 3.05 vs. 2.11 ± 0.42, *p* = *0.014*). No significant differences were observed for other echocardiographic parameters, as well as HGS, training time, and training intensity. The relative power output in CPET was higher in endurance athletes compared to power athletes (4.65 ± 0.73 vs. 4.31 ± 0.68 W/kg, *p* = *0.026*). Results can be found as Supplementary Table 4.

### Longitudinal sub-sample analysis

Eighty-five participants completed two examinations within the MuCAYA-Study and were analyzed as longitudinal sub-samples (V1 vs. V2). The average time between V1 and V2 was 10.6 ± 2.3 months. The majority of athletes performed sports classified as a mixed type of sport (87%). 7% performed power and 6% endurance type of sports. In addition, significant changes in anthropometric parameters, resting heart rate, and z-scores for SBP and DBP were significantly lower at V2 (Table 4). Regarding echocardiographic parameters, absolute values for LVIDd, LVIDs, IVSd, and LVPWd increased significantly from V1 to V2 (*p* < *0.01*); however, when data were transformed into z-scores, a significant difference persisted for IVSd, only, (0.34 ± 0.77 vs. 0.73 ± 0.72, *p* < *0.001*). For further parameters, a significant increase from V1 to V2 was observed: RWT (+ 9.1%), LVM/BSA (+ 11.1%), and LVM/body height (+ 14.8%, for all *p* < *0.001*). E/A decreased by 13.4% (2.98 ± 4.82 vs. 2.58 ± 0.62, p = 0.008), and no significant change was observed in EF and FS (Table 4).

**TABLE 4:** Results of the longitudinal sub-sample regarding anthropometry, heart rate, blood pressure, pulse wave analysis, data on physical performance and training time, and echocardiographic data.

Anthropometry		Visit Nr.1		Visit Nr.2	*P*-value
	n	mean ± SD	n	mean ± SD	
Age [years]	85	13.71 ± 1.60	85	14.63 ± 1.62	**< 0.001**
Body height [cm]	85	166.03 ± 14.60	85	170.51 ± 13.51	**< 0.001**
Body height z-score	85	0.32 ± 1.23	85	0.31 ± 1.24	0.332
Body mass [kg]	85	53.23 ± 14.12	85	58.64 ± 14.30	**< 0.001**
BMI [kg/m^2^]	85	18.93 ± 2.33	85	19.84 ± 2.46	**< 0.001**
BMI z-score	85	-0.16 ± 0.70	85	-0.02 ± 0.69	**< 0.001**
WHR z-score	81	-0.44 ± 0.86	81	-0.61 ± 0.88	**0.036**
WHtR z-score	81	-0.54 ± 0.62	81	-0.41 ± 0.65	**0.004**
BSA [m^2^]	85	1.56 ± 0.27	85	1.66 ± 0.26	**< 0.001**

**Heart rate and blood pressure**		**Visit Nr.1**		**Visit Nr.2**	***P*-value**
	**n**	**mean ± SD**	**n**	**mean ± SD**	

HR [1/min]	84	66.05 ± 9.06	84	62.62 ± 9.57	**< 0.001**
SBP [mmHg]	84	115.19 ± 7.08	84	115.15 ± 7.90	0.964
SBP z-score	83	0.23 ± 0.87	83	-0.04 ± 0.89	**0.002**
DBP [mmHg]	84	63.88 ± 5.70	84	62.86 ± 5.66	0.119
DBP z-score	83	-0.46 ± 0.87	83	-0.74 ± 0.81	**0.003**

**Pulse wave analysis**		**Visit Nr.1**		**Visit Nr.2**	***P*-value**
	**n**	**mean ± SD**	**n**	**mean ± SD**	

PWV [m/s]	84	4.84 ± 0.38	84	4.92 ± 0.42	0.145
PWV z-score	84	0.46 ± 1.19	84	0.34 ± 1.32	0.185
cSBP [mmHg]	84	103.69 ± 8.88	84	105.07 ± 10.01	0.263
cSBP z-score	84	0.34 ± 1.15	84	0.19 ± 1.25	0.101

**Cardiopulmonary exercise test**		**Visit Nr.1**		**Visit Nr.2**	***P*-value**
	**n**	**mean ± SD**	**n**	**mean ± SD**	

Maximum HR [1/min]	70	188.79 ± 9.81	70	187.11 ± 8.78	0.148
Maximum power output [Watt]	71	234.41 ± 69.67	71	269.89 ± 75.81	**< 0.001**
Relative power output [Watt/kg]	71	4.47 ± 0.56	71	4.66 ± 0.69	**0.004**
Relative VO_2peak_ [ml/min/kg]	67	44.23 ± 7.98	67	46.65 ± 6.12	**0.012**

**Handgrip strength**		**Visit Nr.1**		**Visit Nr.2**	***P*-value**
	**n**	**mean ± SD**	**n**	**mean ± SD**	

Maximum HGS/body mass	72	0.51 ± 0.09	72	0.52 ± 0.10	0.115

**Physical activity questionnaire**		**Visit Nr.1**		**Visit Nr.2**	***P*-value**
	**n**	**mean ± SD**	**n**	**mean ± SD**	

Days of physical activity/week	85	5.16 ± 1.18	85	5.36 ± 1.07	0.090
Main sport: training/week [h]	84	7.88 ± 3.19	84	8.75 ± 3.56	**0.003**
Sports club activity: training/week [h]	85	8.22 ± 3.07	85	9.47 ± 3.39	**< 0.001**
Main sport: MET-hours/week	84	71.69 ± 24.12	84	79.87 ± 28.56	**< 0.001**
Sports club activity: MET-hours/week	85	74.43 ± 22.24	85	86.32 ± 24.71	**< 0.001**

**2D transthoracic echocardiography**		**Visit Nr.1**		**Visit Nr.2**	***P*-value**
	**n**	**mean ± SD**	**n**	**mean ± SD**	

EF [%]	79	67.06 ± 5.38	79	66.18 ± 5.41	0.383
FS [%]	79	37.37 ± 4.32	79	36.76 ± 4.20	0.505
LVIDd [mm]	82	47.93 ± 5.04	82	49.12 ± 5.14	**< 0.001**
LVIDd z-score	82	0.20 ± 0.89	82	0.04 ± 1.13	0.113
LVIDs [mm]	81	30.10 ± 3.79	81	31.11 ± 3.94	**0.011**
LVIDs z-score	81	0.25 ± 0.84	81	0.22 ± 0.99	0.397
IVSd [mm]	82	8.05 ± 1.53	82	9.13 ± 1.35	**< 0.001**
IVSd z-score	82	0.34 ± 0.77	82	0.73 ± 0.72	**< 0.001**
LVPWd [mm]	82	7.94 ± 1.33	82	8.40 ± 1.24	**0.001**
LVPWd z-score	82	0.71 ± 0.73	82	0.76 ± 0.80	0.474
Relative wall thickness	82	0.33 ± 0.05	82	0.36 ± 0.04	**< 0.001**
LVM/BSA [g/m^2^]	82	101.90 ± 19.65	82	113. ± 22.87	**< 0.001**
LVM/body height [g/m]	82	95.99 ± 24.38	82	110.21 ± 27.90	**< 0.001**
E/A	37	2.98 ± 4.82	37	2.58 ± 0.62	**0.008**

BMI, body mass index; WHR, waist-to-hip ratio; WHtR, waist-to-height ratio; BSA, body surface area; HR, heart rate; SBP, systolic blood pressure; DBP, diastolic blood pressure; PWV, pulse wave velocity; cSBP/cDBP, central SBP/DBP. EF, ejection fraction; FS, fractional shortening; LVIDd, left ventricular internal diameter in diastole; LVIDs, left ventricular internal diameter in systole; IVSd, interventricular septal thickness in diastole; LVPWd, left ventricular posterior wall thickness in diastole; RWT, relative wall thickness; LVM/BSA, left ventricular mass/body surface area; LVM/body height, left ventricular mass/body height; E/A, ratio of mitral E- and A-wave. The meaning is that these values are significant results as indicated by a p-value of < 0.05.

Participants performed significantly better at V2 in CPET (+ 4.3% W/kg and + 5.5% VO_2peak_) and had increased their training time (+ 15.2%) and training intensity (+ 16%, for all *p* < 0.001).

## Discussion

The present study investigated cardiac adaptations to exercise in young competitive athletes, in relation to weekly training time and training intensity, as well as to exercise performance. The main results of this study were a significant influence of training intensity and VO_2peak_ on LV diameter, IVSd, and LVM/BSA. Thus, the intensity at which young competitive athletes exercise as well as their peak performance level determines cardiac adaptations – and therefore can be potential target parameters to further investigate or modify in this context. It can be potential target parameters to further investigate or modify in this context. Diastolic function was significantly higher in endurance athletes compared to power athletes, which underlines the traditional view of improved cardiac function by cyclic and aerobic exercise on the one hand. On the other hand, in a longitudinal sub-sample, a reduced diastolic function accompanied by increased RWT and IVSd was observed over the course of a year. This result should be further investigated, especially in a longitudinal setting covering a longer period of time.

The term “athlete’s heart” has been under investigation for over 50 years now—for the longest time in adult athletes, only. That cardiac adaptations to exercise are not an exclusive feature of mature athletes but do also happen in children and adolescents has since been recognized (3, 15, 57). In a comprehensive meta-analysis including over 14,000 young competitive athletes, McClean et al. (3) observed larger cardiac diameters and LV wall thickness in young athletes compared to controls. The higher LVIDd, LVIDs, and IVSd compared to reference values in our sample were in the same range as the results by McClean et al. (3). LV systolic and diastolic function, represented by EF and E/A, were not significantly reduced in our cohort. Whereas Pelliccia et al. (26) refer to studies reporting no differences in athletes and non-active controls with an EF being consistently around 50% (58, 59), we report an EF of 66.61 ± 5.77% that corresponds better with the results of recent studies and points towards an improved systolic function in our cohort (3, 60, 61). The same can be stated for diastolic function with an E/A of 2.46 ± 2.16, which is also similar to findings in the current literature (3, 15, 61, 62). As we observed a significantly increased LVIDs and increased wall thickness accompanied by a preserved EF and E/A, we state a functional cardiac adaptation in young competitive athletes. These results are in line with other studies in young athletes (9, 12, 62).

To be able to sustain high physical demands during endurance exercise, the heart increases its output up to 30–40 l/min, imposing a chronic volume overload on the heart that is the potent stimulus for LV dimensions and LVM to increase to the same extent, defined as eccentric hypertrophy (51, 63–65). During power training, blood pressure, heart rate, and peripheral vascular resistance increases which elicits a concentric adaptation of the cardiac muscle (66). This strict categorization of being either endurance or power type of sports does not apply to most disciplines. Regardless of the type of sports, there is an overlap in training regimes with varying degrees of endurance and power components (25, 26, 67). LV eccentric or concentric remodeling can thus be observed in various kinds of disciplines, regardless of the underlying categorization (68). In our study, eccentric hypertrophy was observed in endurance, power, and mixed sports to the same extent (endurance: 56.9%, power: 56.3%, mixed: 51%). Contrary to other authors, concentric hypertrophy was observed in only a minority of participants, again, in power and mixed athletes to the same extent (power: 6.3% and mixed: 6.6%) and to a lesser percentage in endurance athletes (3.9%). Around 30% of athletes within the three categories had normal LV geometry. These numbers differ from the results of Binnetoglu et al. (13), who observed a higher percentage of concentric remodeling (14.3%) and fewer athletes with eccentric remodeling (28.6%) in athletes of various disciplines. Surprisingly, the highest percentage of athletes with eccentric remodeling in this study was observed in power athletes (39.1% in wrestlers). Results by Sulovic et al. (14) reported results of dynamic and static exercising athletes with eccentric hypertrophy in 79.4% of dynamic exercising athletes. In static exercising athletes, the ratio was nearly balanced (54.05% concentric hypertrophy and 45.95% eccentric hypertrophy). In summary, eccentric hypertrophy was observed in more than 50% of our athletes, regardless of the type of sports they performed. Concentric hypertrophy affected only a minority of athletes. One reason for different results could be a shorter exposure to very intense exercise training over a longer period in our sample. Mean age of our participants was younger compared to other studies (13, 14) as well as training exposure compared to Sulovic et al. (14).

Cardiac adaptations observed in this study could be explained by an independent influence of training intensity on chamber dimensions and LVM/BSA. So far, an influence of training history, the type of sports, sex, age, and genetics has been proven (63, 69), but none of the studies screened assessed athletes’ weekly training intensity. We applied a self-reported physical activity questionnaire that allowed the calculation of MET-h/week as an approximate for participants’ training loads. In general, it is the combination of training intensity and training time that corresponds to VO_2peak_ and exercise performance (70). The same might be true for cardiac adaptations, where a certain intensity threshold has to be reached to elicit cardiac adaptations. However, exercise training below this threshold might not trigger cardiac adaptations, regardless of the weekly training time this exercise training is performed.

Bjerring et al. (62) and Rundqvist et al. (8, 61) assessed young athletes’ VO_2max._ They reported correlations with LV volumes but not with IVSd, as observed in our cohort. The authors did not control parameters for sex, age, SBP, BSA, and training history, which could be potential confounders leading to controversial results. Explaining the association of IVSd and VO_2peak_, we assume that athletes with a certain genetic predisposition better respond to exercise training and thus achieve a higher VO_2peak_, may also be more prone to hypertrophic adaptations, as observed in the higher IVSd (63, 71). Additionally, individuals may choose the type of sports that fits best to their individual genetic profile. In this regard, those who are able sustain higher training intensities may become stronger athletes and show more pronounced cardiac adaptations (69, 72).

Boys had higher values for LVM/BSA (+ 15.3%) and LVM/height (+ 17.6%) than girls, which is in line with the current literature and explained by hormonal influences; thus, higher circulating testosterone levels in boys exceeding female levels up to 15 times (73). The significantly higher LVM/BSA in boys was also observed in two different age categories (<14 and ≥14 years). By implication, this stresses the importance to screen male athletes for cardiac adaptations towards LV hypertrophy, as they might be at higher risk than females.

The comparison between endurance and power athletes resulted in a significantly better diastolic function (E/A) in endurance athletes. Venckunas et al. (6) reported a significantly reduced diastolic function in power athletes compared to basketball players but not compared to endurance runners. Contrary to our findings, no significant differences between endurance and power athletes were reported by Sulovic et al. (14) and Binnetoglu et al. (13). Interindividual differences in training time, training intensity, and overall training history do add to genetic trainability and make a direct comparison of results difficult (69). Further cardiac parameters did not significantly differ between the two groups, which could be due to the young age, a lack of specialization at this age, and the mixed nature of any kind of exercise training, where wrestlers also do aerobic exercises, and runners do power training (17, 68). The more specialized training regimes are and the longer they are followed, the more pronounced cardiac adaptations are. In this regard, baseline data are important to further monitor athletes throughout their careers.

A smaller sub-sample of our cohort was examined twice over one year. We observed a lowering of the diastolic function with a 14.5% decrease in E/A that was accompanied by an increase in LVM/BSA (+11.1%), LVM/height (+14.8%), and IVSd (+13.4%). Only three longitudinal studies that assessed echocardiographic parameters in young athletes could be identified. D’Ascenzi et al. (15, 16) examined swimmers during an exercise period of 5 months and reported significantly increased RV and right atrial dimensions, while RV and biatrial function were preserved. Bjerring et al. (74) followed *n* = 36 cross-country skiers over six years. From baseline to the first follow up, participants underwent eccentric cardiac remodeling with significantly increased LV volumes and a reduction in RWT from age 12 to 15, whereas from age 15 to 18 concentric remodeling was observed with a significant higher increase in IVSd, LVPWd, LVM, and RV area. Weekly training time significantly influenced the increase in LVM and IVSd. Furthermore, a non-significant trend toward a reduction in systolic and diastolic LV function was observed. As an adverse consequence of cardiac remodeling, ventricular arrhythmia was observed in senior athletes associated with a longer duration of exercise training (17–19) and a higher risk of tricuspid regurgitation in athletes vs. controls (21). Pelliccia et al. (20) observed the association between adaptations of the LV and left atrium with an LV increase by one mm that was accompanied by a 0.4 mm increase in LA diameter. As an adverse consequence, atrial flutter or fibrillation could occur over time.

Our results confirm the hypothesis that cardiac adaptations to exercise do happen already at a young age in young competitive athletes. In our sample, cardiac adaptation was influenced by exercise intensity and maximum aerobic capacity. These results emphasize how important it is to screen young competitive athletes regularly and to monitor cardiac structure and function in response to exercise training. Parameters in children and adolescents should be compared to sex- and age-adjusted z-scores to better define if values are still within the normal range or exceed the upper limit of normal, thus pointing towards a pathological adaptation. To prevent these adverse adaptations in children and adolescents, a closer observation is needed – especially in a longitudinal setting.

The limitations of this study are the focus on the echocardiographic assessment of LV structure and function, whereas the athlete’s heart does not consist of an altered left ventricle, only. Cardiac adaptations to exercise also affect the right ventricle as well as both atria (15, 19). Pelliccia et al. (26) define a consistent increase in all chambers of the heart as a harmonic adaptation, whereas an inconsistent, e.g., non-harmonic increase, would rather be associated with a non-physiological process. Recent methods like 2D speckle tracking echocardiography or 3D echocardiography would add to better assess the athlete’s heart (60, 62). To better determine training intensity, self-reported activity questionnaires like the one we applied would be the minimum requirement for future studies. Due to technical advances, digital monitoring of training loads via heart rate monitors or smart watches could be easily realized. Most importantly, longitudinal studies are needed with a focus on boys and girls to the same extent and on different training stimuli imposed by different types of sports. We calculated LVM according to the formula of Devereux and Reicheck, published in 1977 (53), which overestimated LVM. The same authors published another formula in 1986 (75), which is recommended by Lang et al. (51) and should be applied in future studies to determine LVM to the latest standards.

## Data availability statement

The raw data supporting the conclusions of this article will be made available by the authors, without undue reservation.

## Ethics statement

This work was part of the Munich Cardiovascular Adaptations in Young Athletes Study (MuCAYA-Study) (24), which was conducted from September 2018 to September 2020 at the Chair of Preventive Pediatrics, TUM Department of Sport and Health Sciences, Technical University of Munich (TUM). This study was approved by the local ethics committee (301/18S) and is in line with the Declaration of Helsinki (2013). Written informed consent was obtained by all participants and legal guardians.

## Author contributions

HW contributed to the study concept and funding, analyzed the data, and drafted the manuscript. LB contributed to the study concept, funding, and reviewed the manuscript. FM and NS performed echocardiographic measurements and reviewed the manuscript. RO-F contributed to the study concept and reviewed the manuscript. All authors contributed to the article and approved the submitted version.
