# Supplementary material for: Training intensity influences left ventricular dimensions in young competitive athletes
Source: Front Cardiovasc Med. 2022 Oct 6;9:961979. doi: 10.3389/fcvm.2022.961979 (PMC9582149; doi:10.3389/fcvm.2022.961979)
Supplement: Supplementary file 1 [file Table_1.docx]

Supplementary Table 1. Types of different sports that are represented in the MuCAYA-Study.

| **Main type of sports** | **all** |  | **males** |  | **females** |  |
| --- | --- | --- | --- | --- | --- | --- |
|  | **n = 404** | **%** | **n = 307** | **%** | **n = 97** | **%** |
| American Football |  |  |  |  |  |  |
| Athletic training | 1 | 0.2 |  |  | 1 | 1.0 |
| Badminton | 1 | 0.2 |  |  | 1 | 1.0 |
| Basketball | 17 | 4.2 | 15 | 4.9 | 1 | 1.0 |
| Biathlon | 3 | 0.7 | 2 | 0.7 | 1 | 1.0 |
| Billard | 5 | 1.2 | 2 | 0.7 | 3 | 3.1 |
| Climbing | 2 | 0.5 | 1 | 0.3 | 1 | 1.0 |
| Cross Country skiing | 23 | 5.7 | 9 | 2.9 | 14 | 14.4 |
| Cycle ball | 1 | 0.2 | 1 | 0.3 |  | 0.0 |
| Cycling | 2 | 0.5 | 1 | 0.3 | 1 | 1.0 |
| Downhill skiing | 6 | 1.5 | 4 | 1.3 | 2 | 2.1 |
| Field hockey | 36 | 8.9 | 19 | 6.2 | 17 | 17.5 |
| Floorball | 1 | 0.2 | 1 | 0.3 |  | 0.0 |
| Gymnastics | 3 | 0.7 | 1 | 0.3 | 2 | 2.1 |
| Handball | 15 | 3.7 | 12 | 3.9 | 3 | 3.1 |
| Hockey | 6 | 1.5 | 6 | 2.0 |  | 0.0 |
| Jiu Jitsu | 4 | 1.0 | 1 | 0.3 | 3 | 3.1 |
| Judo | 4 | 1.0 | 4 | 1.3 |  | 0.0 |
| Kickboxing | 1 | 0.2 | 1 | 0.3 |  | 0.0 |
| Rowing | 20 | 5.0 | 11 | 3.6 | 9 | 9.3 |
| Rugby | 1 | 0.2 |  |  | 1 | 1.0 |
| Running | 3 | 0.7 | 2 | 0.7 | 1 | 1.0 |
| Sailing | 1 | 0.2 | 1 | 0.3 |  | 0.0 |
| Ski jumping | 2 | 0.5 | 2 | 0.7 |  | 0.0 |
| Snowboarding | 1 | 0.2 | 0 | 0.0 | 1 | 1.0 |
| Soccer | 147 | 36.4 | 146 | 47.6 | 1 | 1.0 |
| Swimming | 18 | 4.5 | 10 | 3.3 | 8 | 8.2 |
| Synchronized swimming | 2 | 0.5 |  |  | 2 | 2.1 |
| Taekwondo | 1 | 0.2 |  |  | 1 | 1.0 |
| Track and field | 4 | 1.0 | 2 | 0.7 | 2 | 2.1 |
| Volleyball | 40 | 9.9 | 29 | 9.4 | 11 | 11.3 |
| Wrestling | 30 | 7.4 | 22 | 7.2 | 8 | 8.2 |
| Missing data | 3 | 0.7 | 2 | 0.7 | 1 | 1.0 |
|  |  | 0 |  |  |  |  |
