# Supplementary material for: Training intensity influences left ventricular dimensions in young competitive athletes
Source: Front Cardiovasc Med. 2022 Oct 6;9:961979. doi: 10.3389/fcvm.2022.961979 (PMC9582149; doi:10.3389/fcvm.2022.961979)
Supplement: Supplementary file 2 [file Table_2.docx]

Supplemental Table 2. Echocardiographic parameters for quintiles of training time, training intensity, VO_2peak_, and maximum handgrip strength/ body mass.

|  | **Q1** | | | **Q2** | | | **Q3** | | | **Q4** | | | **Q5** | | |  | |
| --- | --- | --- | --- | --- | --- | --- | --- | --- | --- | --- | --- | --- | --- | --- | --- | --- | --- |
| Dependent variable: **Training time [h/ week]** | males < 5.8 females < 5.4 | | | males 5.8-7  females 5.4-6.8 | | | males 7-8.3 females 6.7-8.9 | | | males 8.3-10.6 females 8.9-11.0 | | | males>10.6 females>11.0 | | | p-value | |
|  |  |  |  |  |  |  |  |  |  |  |  |  |  |  |  |  |  |
| **EF [%]** | 66.92 | ± | 5.21 | 66.58 | ± | 5.25 | 67.26 | ± | 6.23 | 65.85 | ± | 5.73 | 66.16 | ± | 5.70 | .953 |  |
| **FS [%]** | 37.16 | ± | 4.02 | 36.94 | ± | 4.16 | 37.79 | ± | 5.25 | 36.57 | ± | 4.29 | 36.77 | ± | 4.52 | .937 |  |
| **LVIDd [mm]**^1) 2)^ | 46.48 | ± | 5.06 | 47.42 | ± | 3.98 | 48.33 | ± | 4.70 | 49.69 | ± | 4.99 | 49.66 | ± | 4.51 | **.021** |  |
| **LVIDs [mm]** | 29.21 | ± | 3.73 | 29.88 | ± | 3.20 | 30.09 | ± | 3.95 | 31.54 | ± | 4.10 | 31.36 | ± | 3.88 | .226 |  |
| **IVSd [mm]** | 8.10 | ± | 1.36 | 8.66 | ± | 1.57 | 8.51 | ± | 1.37 | 8.55 | ± | 1.24 | 8.71 | ± | 1.46 | .657 |  |
| **LVPWd [mm]** | 7.59 | ± | 1.45 | 7.95 | ± | 1.18 | 8.14 | ± | 1.33 | 7.96 | ± | 1.34 | 8.48 | ± | 1.26 | .103 |  |
| **Relative wall thickness** | 0.34 | ± | 0.06 | 0.35 | ± | 0.05 | 0.35 | ± | 0.05 | 0.33 | ± | 0.04 | 0.35 | ± | 0.04 | .056 |  |
| **LVM/ BSA [g/m^2^]** | 170.92 | ± | 31.10 | 177.76 | ± | 25.38 | 179.65 | ± | 31.15 | 184.26 | ± | 30.85 | 179.65 | ± | 32.08 | .470 |  |
| **E/A** | 2.68 | ± | 3.09 | 2.22 | ± | 0.50 | 2.10 | ± | 0.40 | 2.35 | ± | 0.57 | 3.04 | ± | 4.73 | .281 |  |
| ^1)^ Q1 vs. Q4, p=.024 ^2)^ Q4 vs. Q5, p=.046 |  |  |  |  |  |  |  |  |  |  |  |  |  |  |  |  |  |
|  | | | | | | | | | | | | | | | | | |
|  | **Q1** | | | **Q2** | | | **Q3** | | | **Q4** | | | **Q5** | | |  | |
| Dependent variable: **Training intensity  [MET-hours/ week]** | males<55.0 females<40.7 | | | males 55.0-66.7 females 40.7-58.1 | | | males 66.7-80.8 females 58.1-76.7 | | | males 80.8-100.0 females 76.7-101.5 | | | males > 100.0 females > 101.5 | | | **p-value** | |
|  |  |  |  |  |  |  |  |  |  |  |  |  |  |  |  |  |  |
| **EF [%]** | 67.61 | ± | 6.68 | 67.57 | ± | 5.06 | 67.20 | ± | 5.79 | 65.42 | ± | 4.42 | 65.06 | ± | 5.83 | .170 |  |
| **FS [%]** | 37.96 | ± | 5.62 | 37.73 | ± | 4.11 | 37.47 | ± | 4.53 | 36.20 | ± | 3.42 | 35.97 | ± | 4.39 | .274 |  |
| **LVIDd [mm]** | 47.61 | ± | 5.51 | 47.81 | ± | 4.55 | 47.80 | ± | 4.61 | 48.60 | ± | 4.80 | 49.97 | ± | 4.18 | .125 |  |
| **LVIDs [mm]** | 29.57 | ± | 4.42 | 29.70 | ± | 3.35 | 29.94 | ± | 4.01 | 31.03 | ± | 3.31 | 31.94 | ± | 3.78 | .053 |  |
| **IVSd [mm]** | 8.16 | ± | 1.31 | 8.37 | ± | 1.32 | 8.53 | ± | 1.65 | 8.59 | ± | 1.36 | 8.89 | ± | 1.33 | .489 |  |
| **LVPWd [mm]** | 7.89 | ± | 1.57 | 7.99 | ± | 1.28 | 8.02 | ± | 1.25 | 8.02 | ± | 1.27 | 8.26 | ± | 1.32 | .922 |  |
| **Relative wall thickness** | 0.34 | ± | 0.06 | 0.34 | ± | 0.04 | 0.35 | ± | 0.06 | 0.34 | ± | 0.04 | 0.34 | ± | 0.04 | .744 |  |
| **LVM/ BSA [g/m^2^]** | 173.68 | ± | 32.37 | 179.27 | ± | 27.62 | 173.79 | ± | 31.47 | 181.52 | ± | 30.86 | 184.92 | ± | 28.21 | .245 |  |
| **E/A** | 2.21 | ± | 0.60 | 2.60 | ± | 2.82 | 2.83 | ± | 4.47 | 2.25 | ± | 0.53 | 2.34 | ± | 0.47 | .582 |  |
|  |  |  |  |  |  |  |  |  |  |  |  |  |  |  |  |  |  |
|  | | | | | | | | | | | | | | | | | |
|  | **Q1** | | | **Q2** | | | **Q3** | | | **Q4** | | | **Q5** | | |  | |
| Dependent variable: **VO_2peak_ [ml/min/kg]** | males < 41.2 females < 32.6 | | | males 41.2-44.9 females 32.6-35.6 | | | males 44.9-48 females 35.6-39.3 | | | males 48.0-52.0 females 39.3-43.0 | | | males > 52.0 females > 43.0 | | | **p-value** | |
|  |  |  |  |  |  |  |  |  |  |  |  |  |  |  |  |  |  |
| **EF [%]** | 67.23 | ± | 5.97 | 66.87 | ± | 5.65 | 65.82 | ± | 4.36 | 66.30 | ± | 6.32 | 66.61 | ± | 5.81 | .548 |  |
| **FS [%]** | 37.62 | ± | 5.06 | 37.24 | ± | 4.38 | 36.38 | ± | 3.47 | 36.95 | ± | 4.84 | 37.12 | ± | 4.57 | .508 |  |
| **LVIDd [mm]** | 48.28 | ± | 5.31 | 47.05 | ± | 4.76 | 48.96 | ± | 4.46 | 48.19 | ± | 4.54 | 49.24 | ± | 4.77 | .075 |  |
| **LVIDs [mm]** | 30.13 | ± | 4.75 | 29.60 | ± | 3.79 | 31.11 | ± | 3.14 | 30.37 | ± | 3.54 | 30.93 | ± | 3.95 | .057 |  |
| **IVSd [mm]** | 8.15 | ± | 1.57 | 8.31 | ± | 1.27 | 8.56 | ± | 1.23 | 8.79 | ± | 1.56 | 8.71 | ± | 1.36 | .448 |  |
| **LVPWd [mm]** | 7.73 | ± | 1.35 | 7.94 | ± | 1.34 | 7.83 | ± | 1.24 | 8.31 | ± | 1.47 | 8.30 | ± | 1.23 | .162 |  |
| **Relative wall thickness** | 0.33 | ± | 0.06 | 0.35 | ± | 0.04 | 0.34 | ± | 0.05 | 0.36 | ± | 0.05 | 0.35 | ± | 0.04 | .215 |  |
| **LVM/ BSA [g/m^2^]** | 174.77 | ± | 29.20 | 170.27 | ± | 31.66 | 180.33 | ± | 26.56 | 181.47 | ± | 33.22 | 185.92 | ± | 28.97 | .140 |  |
| **E/A** | 2.16 | ± | 0.51 | 2.90 | ± | 4.26 | 2.26 | ± | 0.61 | 2.62 | ± | 2.86 | 2.24 | ± | 0.42 | .659 |  |
|  |  |  |  |  |  |  |  |  |  |  |  |  |  |  |  |  |  |
|  | | | | | | | | | | | | | | | | | |
|  | **Q1** | | | **Q2** | | | **Q3** | | | **Q4** | | | **Q5** | | |  | |
| Dependent variable: **Maximum HGS/ body mass** | males < 0.45 females < 0.44 | | | males 0.45-0.51 females 0.44-0.47 | | | males 0.51-0.56 females 0.47-0.52 | | | males 0.56-0.62 females 0.52-0.56 | | | males > 0.62 females > 0.56 | | | **p-value** | |
|  |  |  |  |  |  |  |  |  |  |  |  |  |  |  |  |  |  |
| **EF [%]** | 67.57 | ± | 6.29 | 67.26 | ± | 5.44 | 66.31 | ± | 5.41 | 66.38 | ± | 5.57 | 65.21 | ± | 5.25 | .680 |  |
| **FS [%]** | 37.74 | ± | 5.25 | 37.66 | ± | 4.30 | 36.91 | ± | 4.04 | 36.94 | ± | 4.35 | 36.00 | ± | 4.20 | .586 |  |
| **LVIDd [mm]** | 45.65 | ± | 4.41 | 47.89 | ± | 4.36 | 49.28 | ± | 4.99 | 49.25 | ± | 4.90 | 49.78 | ± | 4.17 | .503 |  |
| **LVIDs [mm]** | 28.47 | ± | 4.05 | 29.77 | ± | 3.13 | 31.07 | ± | 3.85 | 31.16 | ± | 4.02 | 31.76 | ± | 3.32 | .689 |  |
| **IVSd [mm]** | 8.25 | ± | 1.65 | 8.16 | ± | 1.18 | 8.56 | ± | 1.40 | 8.65 | ± | 1.35 | 8.95 | ± | 1.31 | .484 |  |
| **LVPWd [mm]** | 7.50 | ± | 1.40 | 7.77 | ± | 1.12 | 8.15 | ± | 1.43 | 8.38 | ± | 1.33 | 8.37 | ± | 1.18 | .676 |  |
| **Relative wall thickness** | 0.35 | ± | 0.06 | 0.33 | ± | 0.04 | 0.34 | ± | 0.05 | 0.35 | ± | 0.05 | 0.35 | ± | 0.04 | .407 |  |
| **LVM/ BSA [g/m^2^]** | 169.91 | ± | 28.44 | 176.96 | ± | 28.72 | 179.98 | ± | 30.04 | 182.12 | ± | 31.85 | 184.58 | ± | 31.31 | 990 |  |
| **E/A** | 2.95 | ± | 4.26 | 2.32 | ± | 0.57 | 2.18 | ± | 0.43 | 2.60 | ± | 3.03 | 2.17 | ± | 0.43 | .472 |  |
