# Supplementary material for: Training intensity influences left ventricular dimensions in young competitive athletes
Source: Front Cardiovasc Med. 2022 Oct 6;9:961979. doi: 10.3389/fcvm.2022.961979 (PMC9582149; doi:10.3389/fcvm.2022.961979)
Supplement: Supplementary file 3 [file Table_3.docx]

Supplemental Table 3. Anthropometric data, heart rate and blood pressure, pulse wave analysis, cardiopulmonary exercise testing, maximum handgrip strength/ body mass, physical activity questionnaire, and echocardiographic data for boys and girls < 14 years and > 14 years.

|  |  | **AGE < 14 years** | | | | | | |  |  |  | **AGE > 14 years** | | | | | | |  |
| --- | --- | --- | --- | --- | --- | --- | --- | --- | --- | --- | --- | --- | --- | --- | --- | --- | --- | --- | --- |
|  |  |  |  |  |  |  |  |  |  |  |  |  |  |  |  |  |  |  |  |
| **Anthropometry** |  | **males** | | |  | **females** | | | **p-value** |  |  | **males** | | |  | **females** | | | **p-value** |
|  | **n** | **mean ± SD** | | | **n** | **mean ± SD** | | |  |  | **n** | **mean ± SD** | | | **n** | **mean ± SD** | | |  |
|  |  |  |  |  |  |  |  |  |  |  |  |  |  |  |  |  |  |  |  |
| Age [years] | 124 | 12.27 | ± | 1.30 | 38 | 12.13 | ± | 1.62 | .583 |  | 183 | 15.62 | ± | 1.00 | 59 | 15.38 | ± | 1.00 | .113 |
| Body height [cm] | 124 | 155.75 | ± | 11.08 | 38 | 155.10 | ± | 12.14 | .759 |  | 182 | 177.42 | ± | 10.26 | 59 | 167.15 | ± | 7.27 | **<.001** |
| Body height z-score | 124 | 0.12 | ± | 0.91 | 38 | 0.20 | ± | 1.21 | .644 |  | 182 | 0.41 | ± | 1.28 | 59 | 0.39 | ± | 1.12 | .934 |
| Body mass [kg] | 124 | 44.27 | ± | 9.77 | 38 | 46.28 | ± | 12.90 | .308 |  | 182 | 66.77 | ± | 11.61 | 59 | 57.91 | ± | 7.97 | **<.001** |
| BMI [kg/m^2^] | 124 | 18.03 | ± | 2.16 | 38 | 18.86 | ± | 3.39 | .162 |  | 182 | 21.08 | ± | 2.42 | 59 | 20.67 | ± | 1.93 | .237 |
| BMI z-score | 124 | -0.19 | ± | 0.84 | 38 | -0.01 | ± | 1.00 | .264 |  | 182 | 0.19 | ± | 0.73 | 59 | 0.05 | ± | 0.65 | .197 |
| WHR z-score | 111 | -0.52 | ± | 1.09 | 29 | -0.26 | ± | 0.96 | .242 |  | 182 | -0.45 | ± | 1.05 | 59 | -0.12 | ± | 1.09 | **.042** |
| WHtR z-score | 111 | -0.56 | ± | 0.71 | 29 | -0.31 | ± | 0.92 | .111 |  | 182 | -0.30 | ± | 0.70 | 59 | -0.41 | ± | 0.60 | .268 |
| BSA [m^2^] | 124 | 1.38 | ± | 0.20 | 38 | 1.40 | ± | 0.25 | .594 |  | 182 | 1.81 | ± | 0.20 | 59 | 1.64 | ± | 0.14 | **<.001** |
|  |  |  |  |  |  |  |  |  |  |  |  |  |  |  |  |  |  |  |  |
| **Heart rate and blood pressure** |  | **males** | | |  | **females** | | | **p-value** |  |  | **males** | | |  | **females** | | | **p-value** |
|  | **n** | **mean ± SD** | | | **n** | **mean ± SD** | | |  |  | **n** | **mean ± SD** | | | **n** | **mean ± SD** | | |  |
|  |  |  |  |  |  |  |  |  |  |  |  |  |  |  |  |  |  |  |  |
| HR [1/min] | 124 | 66.22 | ± | 8.37 | 37 | 71.24 | ± | 11.59 | .018 |  | 183 | 63.68 | ± | 10.10 | 59 | 63.68 | ± | 10.99 | .999 |
| SBP [mmHg] | 124 | 113.49 | ± | 7.99 | 37 | 112.70 | ± | 7.00 | .563 |  | 183 | 119.76 | ± | 9.23 | 59 | 112.71 | ± | 6.86 | **<.001** |
| SBP z-score | 124 | 0.54 | ± | 0.86 | 37 | 0.47 | ± | 0.73 | .685 |  | 182 | 0.06 | ± | 0.90 | 59 | -0.05 | ± | 0.83 | .395 |
| DBP [mmHg] | 124 | 63.57 | ± | 6.42 | 37 | 63.70 | ± | 6.05 | .913 |  | 183 | 63.44 | ± | 6.21 | 59 | 63.31 | ± | 6.02 | .882 |
| DBP z-score | 124 | -0.29 | ± | 0.93 | 37 | -0.26 | ± | 0.93 | .893 |  | 182 | -0.81 | ± | 0.85 | 59 | -0.76 | ± | 0.89 | .727 |
|  |  |  |  |  |  |  |  |  |  |  |  |  |  |  |  |  |  |  |  |
| **Pulse wave analysis** |  | **males** | | |  | **females** | | | **p-value** |  |  | **males** | | |  | **females** | | | **p-value** |
|  | **n** | **mean ± SD** | | | **n** | **mean ± SD** | | |  |  | **n** | **mean ± SD** | | | **n** | **mean ± SD** | | |  |
|  |  |  |  |  |  |  |  |  |  |  |  |  |  |  |  |  |  |  |  |
| PWV [m/s] | 124 | 4.69 | ± | 0.36 | 37 | 4.64 | ± | 0.29 | .435 |  | 183 | 5.11 | ± | 0.44 | 59 | 4.83 | ± | 0.35 | **<.001** |
| PWV z-score | 122 | 0.57 | ± | 1.37 | 36 | 0.50 | ± | 0.96 | .758 |  | 183 | 0.46 | ± | 1.38 | 59 | 0.42 | ± | 1.45 | .842 |
| cSBP [mmHg] | 124 | 100.31 | ± | 8.83 | 37 | 100.35 | ± | 8.76 | .978 |  | 183 | 109.60 | ± | 10.15 | 59 | 104.41 | ± | 8.54 | **<.001** |
| cSBP z-score | 122 | 0.46 | ± | 1.26 | 36 | 0.50 | ± | 1.19 | .873 |  | 183 | 0.37 | ± | 1.34 | 59 | 0.26 | ± | 1.27 | .588 |
|  |  |  |  |  |  |  |  |  |  |  |  |  |  |  |  |  |  |  |  |
| **Cardiopulmonary exercise test** |  | **males** | | |  | **females** | | | **p-value** |  |  | **males** | | |  | **females** | | | **p-value** |
|  | **n** | **mean ± SD** | | | **n** | **mean ± SD** | | |  |  | **n** | **mean ± SD** | | | **n** | **mean ± SD** | | |  |
|  |  |  |  |  |  |  |  |  |  |  |  |  |  |  |  |  |  |  |  |
| Maximum HR [1/min] | 118 | 188.84 | ± | 10.39 | 32 | 185.09 | ± | 13.32 | .092 |  | 164 | 184.68 | ± | 11.28 | 47 | 188.53 | ± | 8.95 | **.032** |
| Maximum power output [Watt] | 118 | 195.39 | ± | 49.84 | 32 | 186.75 | ± | 48.01 | .382 |  | 164 | 314.70 | ± | 55.67 | 49 | 236.43 | ± | 37.01 | **<.001** |
| Relative power output [Watt/kg] | 118 | 4.37 | ± | 0.56 | 32 | 4.00 | ± | 0.75 | **.002** |  | 164 | 4.76 | ± | 0.52 | 49 | 4.06 | ± | 0.55 | **<.001** |
| Relative VO2peak [ml/min/kg] | 118 | 44.52 | ± | 5.96 | 31 | 36.31 | ± | 7.38 | **<.001** |  | 160 | 47.23 | ± | 6.93 | 49 | 38.71 | ± | 5.97 | **<.001** |
|  |  |  |  |  |  |  |  |  |  |  |  |  |  |  |  |  |  |  |  |
| **Handgrip strength** |  | **males** | | |  | **females** | | | **p-value** |  |  | **males** | | |  | **females** | | | **p-value** |
|  | **n** | **mean ± SD** | | | **n** | **mean ± SD** | | |  |  | **n** | **mean ± SD** | | | **n** | **mean ± SD** | | |  |
|  |  |  |  |  |  |  |  |  |  |  |  |  |  |  |  |  |  |  |  |
| Maximum HGS/ body mass | 112 | 0.47 | ± | 0.09 | 34 | 0.46 | ± | 0.08 | .523 |  | 170 | 0.58 | ± | 0.08 | 50 | 0.52 | ± | 0.07 | **<.001** |
|  |  |  |  |  |  |  |  |  |  |  |  |  |  |  |  |  |  |  |  |
| **Physical activity questionnaire** |  | **males** | | |  | **females** | | | **p-value** |  |  | **males** | | |  | **females** | | | **p-value** |
|  | **n** | **mean ± SD** | | | **n** | **mean ± SD** | | |  |  | **n** | **mean ± SD** | | | **n** | **mean ± SD** | | |  |
|  |  |  |  |  |  |  |  |  |  |  |  |  |  |  |  |  |  |  |  |
| Days of physical activity/ week | 124 | 5.24 | ± | 1.19 | 38 | 4.95 | ± | 1.39 | .203 |  | 183 | 5.30 | ± | 1.16 | 59 | 4.91 | ± | 1.14 | **.023** |
| Main sport: training/ week [h] | 124 | 6.67 | ± | 2.95 | 38 | 6.70 | ± | 3.31 | .951 |  | 183 | 8.96 | ± | 3.54 | 59 | 8.39 | ± | 4.50 | .312 |
| Sports club activity: training/ week [h] | 124 | 7.27 | ± | 2.93 | 38 | 7.59 | ± | 3.15 | .560 |  | 183 | 9.19 | ± | 3.69 | 59 | 8.85 | ± | 4.12 | .559 |
| Main sport: MET-hours/ week | 123 | 64.09 | ± | 26.88 | 37 | 54.43 | ± | 31.19 | .067 |  | 181 | 80.61 | ± | 35.65 | 59 | 74.61 | ± | 49.74 | .393 |
| Sports club activity: MET-hours/ week | 124 | 70.61 | ± | 23.63 | 37 | 65.01 | ± | 31.18 | .318 |  | 182 | 85.10 | ± | 33.94 | 59 | 82.12 | ± | 45.50 | .644 |
|  |  |  |  |  |  |  |  |  |  |  |  |  |  |  |  |  |  |  |  |
| **2D transthoracic echocardiography** |  | **males** | | |  | **females** | | | **p-value** |  |  | **males** | | |  | **females** | | | **p-value** |
|  | **n** | **mean ± SD** | | | **n** | **mean ± SD** | | |  |  | **n** | **mean ± SD** | | | **n** | **mean ± SD** | | |  |
|  |  |  |  |  |  |  |  |  |  |  |  |  |  |  |  |  |  |  |  |
| EF [%] | 119 | 67.77 | ± | 5.12 | 37 | 69.19 | ± | 6.26 | .166 |  | 175 | 65.49 | ± | 5.99 | 58 | 65.98 | ± | 5.22 | .577 |
| FS [%] | 119 | 37.75 | ± | 4.07 | 37 | 39.16 | ± | 5.54 | .094 |  | 174 | 36.43 | ± | 4.74 | 58 | 36.50 | ± | 4.19 | .915 |
| LVIDd [mm] | 119 | 45.27 | ± | 3.60 | 37 | 44.41 | ± | 3.99 | .221 |  | 175 | 51.13 | ± | 4.39 | 58 | 47.48 | ± | 3.62 | **<.001** |
| LVIDd z-score | 119 | 0.34 | ± | 0.56 | 37 | 0.07 | ± | 0.62 | **.016** |  | 174 | -0.12 | ± | 1.13 | 58 | 0.14 | ± | 0.65 | .091 |
| LVIDs [mm] | 119 | 28.20 | ± | 2.90 | 37 | 27.02 | ± | 3.26 | .038 |  | 174 | 32.42 | ± | 3.78 | 58 | 30.17 | ± | 3.14 | **<.001** |
| LVIDs z-score | 119 | 0.28 | ± | 0.64 | 37 | -0.12 | ± | 0.96 | **.003** |  | 173 | 0.10 | ± | 0.99 | 58 | 0.29 | ± | 0.79 | .136 |
| IVSd [mm] | 119 | 8.04 | ± | 1.45 | 37 | 7.80 | ± | 1.31 | .371 |  | 177 | 9.17 | ± | 1.29 | 58 | 8.26 | ± | 1.20 | **<.001** |
| IVSd z-score | 119 | 0.72 | ± | 0.72 | 37 | 0.54 | ± | 0.67 | .194 |  | 176 | 0.45 | ± | 0.68 | 58 | 0.38 | ± | 0.76 | .523 |
| LVPWd [mm] | 119 | 7.40 | ± | 1.14 | 37 | 7.41 | ± | 1.42 | .993 |  | 175 | 8.81 | ± | 1.15 | 58 | 7.66 | ± | 1.41 | **<.001** |
| LVPWd z-score | 119 | 0.81 | ± | 0.68 | 37 | 0.74 | ± | 0.81 | .560 |  | 174 | 0.64 | ± | 0.78 | 58 | 0.41 | ± | 0.95 | .069 |
| Relative wall thickness | 119 | 0.34 | ± | 0.05 | 37 | 0.34 | ± | 0.05 | .905 |  | 175 | 0.35 | ± | 0.05 | 58 | 0.34 | ± | 0.06 | .066 |
| LVM/ BSA [g/m^2^] | 119 | 170.67 | ± | 25.99 | 37 | 159.58 | ± | 22.14 | **.020** |  | 174 | 193.29 | ± | 30.89 | 58 | 163.51 | ± | 25.97 | **<.001** |
| LVM/ body height [g/m] | 119 | 88.47 | ± | 20.17 | 37 | 84.48 | ± | 20.01 | .294 |  | 174 | 116.58 | ± | 22.12 | 58 | 92.52 | ± | 18.53 | **<.001** |
| E/A | 79 | 2.69 | ± | 3.31 | 29 | 2.95 | ± | 3.64 | .726 |  | 126 | 2.22 | ± | 0.50 | 44 | 2.39 | ± | 0.54 | .061 |
| BMI = body mass index, WHR = waist-to-hip ratio, WHtR = waist-to-height ratio, BSA = body surface area, HR = heart rate, SBP = systolic blood pressure, DBP = diastolic blood pressure, PWV = pulse wave velocity, cSBP/ cDBP = central SBP/ DBP. EF = ejection fraction, FS = fractional shortening, LVIDd = left ventricular internal diameter in diastole, LVIDs = left ventricular internal diameter in systole, IVSd = interventricular septal thickness in diastole, LVPWd = left ventricular posterior wall thickness in diastole, RWT = relative wall thickness, LVM/ BSA = left ventricular mass/ body surface area, LVM/ body height = left ventricular mass/ body height, E/A = ratio of mitral E- and A-wave. | | | | | | | | | | | | | | | | | | | |
