# Supplementary material for: Training intensity influences left ventricular dimensions in young competitive athletes
Source: Front Cardiovasc Med. 2022 Oct 6;9:961979. doi: 10.3389/fcvm.2022.961979 (PMC9582149; doi:10.3389/fcvm.2022.961979)
Supplement: Supplementary file 4 [file Table_4.docx]

Supplemental Table 4. Anthropometric data, heart rate and blood pressure, pulse wave analysis, cardiopulmonary exercise testing, maximum handgrip strength/ body mass, physical activity questionnaire, and echocardiographic data for athletes performing endurance and power type of sports.

| **Anthropometry** |  | **endurance** | | |  | **power** | | | **p-value** |
| --- | --- | --- | --- | --- | --- | --- | --- | --- | --- |
|  | **n** | **mean ± SD** | | | **n** | **mean ± SD** | | |  |
|  |  |  |  |  |  |  |  |  |  |
| Age [years] | 51 | 14.42 | ± | 1.83 | 48 | 14.67 | ± | 1.70 | .481 |
| Body height [cm] | 51 | 165.98 | ± | 12.77 | 47 | 165.51 | ± | 11.98 | .852 |
| Body height z-score | 51 | 0.24 | ± | 1.03 | 47 | -0.29 | ± | 1.07 | **.015** |
| Body mass [kg] | 51 | 56.21 | ± | 13.88 | 47 | 57.66 | ± | 14.77 | .617 |
| BMI [kg/m^2^] | 51 | 20.10 | ± | 2.85 | 47 | 20.71 | ± | 3.16 | .504 |
| BMI z-score | 51 | 0.03 | ± | 0.91 | 47 | 0.20 | ± | 0.87 | .351 |
| WHR z-score | 49 | -0.24 | ± | 0.95 | 45 | -0.33 | ± | 1.28 | .686 |
| WHtR z-score | 49 | -0.33 | ± | 0.82 | 45 | -0.20 | ± | 0.67 | .390 |
| BSA [m^2^] | 51 | 1.60 | ± | 0.26 | 47 | 1.62 | ± | 0.26 | .744 |
|  |  |  |  |  |  |  |  |  |  |
|  |  |  |  |  |  |  |  |  |  |
| **Heart rate and blood pressure** |  | **endurance** | | |  | **power** | | | **p-value** |
|  | **n** | **mean ± SD** | | | **n** | **mean ± SD** | | |  |
|  |  |  |  |  |  |  |  |  |  |
| HR [1/min] | 51 | 66.04 | ± | 12.20 | 48 | 68.54 | ± | 12.21 | .310 |
| SBP [mmHg] | 51 | 115.08 | ± | 10.92 | 48 | 118.58 | ± | 9.46 | .092 |
| SBP z-score | 51 | 0.16 | ± | 1.04 | 47 | 0.49 | ± | 0.88 | .091 |
| DBP [mmHg] | 51 | 62.06 | ± | 5.81 | 48 | 66.08 | ± | 8.08 | **.006** |
| DBP z-score | 51 | -0.81 | ± | 0.82 | 47 | -0.23 | ± | 1.20 | **.007** |
|  |  |  |  |  |  |  |  |  |  |
|  |  |  |  |  |  |  |  |  |  |
| **Pulse wave analysis** |  | **endurance** | | |  | **power** | | | **p-value** |
|  | **n** | **mean ± SD** | | | **n** | **mean ± SD** | | |  |
|  |  |  |  |  |  |  |  |  |  |
| PWV [m/s] | 51 | 4.85 | ± | 0.50 | 48 | 4.97 | ± | 0.46 | .168 |
| PWV z-score | 51 | 0.39 | ± | 1.58 | 48 | 0.59 | ± | 1.39 | .512 |
| cSBP [mmHg] | 51 | 103.55 | ± | 12.04 | 48 | 107.67 | ± | 11.61 | .086 |
| cSBP z-score | 51 | 0.21 | ± | 1.50 | 48 | 0.62 | ± | 1.55 | .182 |
|  |  |  |  |  |  |  |  |  |  |
|  |  |  |  |  |  |  |  |  |  |
| **2D transthoracic echocardiography** |  | **endurance** | | |  | **power** | | | **p-value** |
|  | **n** | **mean ± SD** | | | **n** | **mean ± SD** | | |  |
|  |  |  |  |  |  |  |  |  |  |
| EF [%] | 48 | 66.52 | ± | 5.74 | 48 | 65.25 | ± | 5.16 | .256 |
| FS [%] | 48 | 37.06 | ± | 4.46 | 48 | 35.88 | ± | 3.99 | .215 |
| LVIDd [mm] | 48 | 48.48 | ± | 4.76 | 48 | 48.05 | ± | 5.33 | .670 |
| LVIDd z-score | 48 | 0.22 | ± | 0.86 | 47 | 0.08 | ± | 0.86 | .319 |
| LVIDs [mm] | 48 | 30.59 | ± | 4.09 | 48 | 30.73 | ± | 4.16 | .873 |
| LVIDs z-score | 48 | 0.30 | ± | 0.90 | 47 | 0.30 | ± | 0.81 | .963 |
| IVSd [mm] | 48 | 8.47 | ± | 1.45 | 48 | 8.55 | ± | 1.26 | .769 |
| IVSd z-score | 48 | 0.51 | ± | 0.79 | 47 | 0.56 | ± | 0.66 | .721 |
| LVPWd [mm] | 48 | 7.83 | ± | 1.29 | 48 | 8.25 | ± | 1.25 | .238 |
| LVPWd z-score | 48 | 0.56 | ± | 0.85 | 47 | 0.81 | ± | 0.86 | .159 |
| Relative wall thickness | 48 | 0.34 | ± | 0.05 | 48 | 0.35 | ± | 0.05 | .128 |
| LVM/ BSA [g/m^2^] | 48 | 179.16 | ± | 31.60 | 47 | 180.34 | ± | 35.25 | .864 |
| LVM/ body height [g/m] | 48 | 100.59 | ± | 24.80 | 47 | 104.51 | ± | 25.70 | .451 |
| E/A | 41 | 2.87 | ± | 3.05 | 33 | 2.11 | ± | 0.42 | **.014** |
|  |  |  |  |  |  |  |  |  |  |
|  |  |  |  |  |  |  |  |  |  |
| **Cardiopulmonary exercise test** |  | **endurance** | | |  | **power** | | | **p-value** |
|  | **n** | **mean ± SD** | | | **n** | **mean ± SD** | | |  |
|  |  |  |  |  |  |  |  |  |  |
| Maximum HR [1/min] | 46 | 189.46 | ± | 8.35 | 43 | 186.07 | ± | 14.60 | .375 |
| Maximum power output [Watt] | 47 | 264.91 | ± | 80.74 | 43 | 245.33 | ± | 68.09 | .232 |
| Relative power output [Watt/kg] | 47 | 4.65 | ± | 0.73 | 43 | 4.31 | ± | 0.68 | **.026** |
| Relative VO_2peak_ [ml/min/kg] | 46 | 44.68 | ± | 9.16 | 42 | 42.82 | ± | 5.74 | .254 |
|  |  |  |  |  |  |  |  |  |  |
|  |  |  |  |  |  |  |  |  |  |
| **Handgrip strength** |  | **endurance** | | |  | **power** | | | **p-value** |
|  | **n** | **mean ± SD** | | | **n** | **mean ± SD** | | |  |
|  |  |  |  |  |  |  |  |  |  |
| Maximum HGS/ body mass | 46 | 0.53 | ± | 0.11 | 47 | 0.56 | ± | 0.08 | .098 |
|  |  |  |  |  |  |  |  |  |  |
|  |  |  |  |  |  |  |  |  |  |
| **Physical activity questionnaire** |  | **endurance** | | |  | **power** | | | **p-value** |
|  | **n** | **mean ± SD** | | | **n** | **mean ± SD** | | |  |
|  |  |  |  |  |  |  |  |  |  |
| Days of physical activity/ week | 51 | 5.17 | ± | 1.13 | 48 | 4.89 | ± | 1.14 | .220 |
| Main sport: training/ week [h] | 50 | 382.40 | ± | 181.06 | 48 | 409.08 | ± | 208.97 | .839 |
| Sports club activity: training/ week [h] | 51 | 457.39 | ± | 163.30 | 48 | 505.68 | ± | 233.73 | .522 |
| Main sport: MET-hours/ week | 50 | 66.44 | ± | 37.23 | 48 | 74.48 | ± | 45.09 | .493 |
| Sports club activity: MET-hours/ week | 51 | 76.45 | ± | 33.14 | 48 | 86.42 | ± | 40.95 | .261 |
|  |  |  |  |  |  |  |  |  |  |
|  |  |  |  |  |  |  |  |  |  |
| BMI = body mass index, WHR = waist-to-hip ratio, WHtR = waist-to-height ratio, BSA = body surface area,  HR = heart rate, SBP = systolic blood pressure, DBP = diastolic blood pressure, PWV = pulse wave velocity, cSBP/ cDBP = central SBP/ DBP. EF = ejection fraction, FS = fractional shortening, LVIDd = left ventricular internal diameter in diastole, LVIDs = left ventricular internal diameter in systole, IVSd = interventricular septal thickness in diastole, LVPWd = left ventricular posterior wall thickness in diastole, RWT = relative wall thickness, LVM/ BSA = left ventricular mass/ body surface area, LVM/ body height = left ventricular mass/ body height, E/A = ratio of mitral E- and A-wave. | | | | | | | | | |
